# Complete Mitochondrial Genome Sequence of the Gulf Coast Tick (Amblyomma maculatum)

**DOI:** 10.1128/MRA.00431-21

**Published:** 2021-09-23

**Authors:** Amanda E. Brenner, Rahul Raghavan

**Affiliations:** a Department of Biology, Portland State University, Portland, Oregon, USA; b Department of Biology, The University of Texas at San Antonio, San Antonio, Texas, USA; University of California, Riverside

## Abstract

The complete circularized mitochondrial genome sequence of Amblyomma maculatum is 14,803 bp long. It encodes 13 protein coding genes, 2 rRNA genes, 22 tRNA genes, 2 tick box motifs, and 2 control regions. The gene arrangement and content are consistent with those of previously reported Metastriata tick mitochondrial genomes.

## ANNOUNCEMENT

While many *Amblyomma* species have established long-term relationships with *Coxiella*-like endosymbionts (CLEs), Amblyomma maculatum contains a *Francisella*-like endosymbiont (FLE) that likely supplements its obligate hematophagous diet with essential vitamins (1–5). In order to understand the dynamics of endosymbiont-tick coevolution, it is necessary to establish the relationships among ticks. In contribution to this effort, in this genome announcement, we describe the complete mitochondrial genome sequence of the Gulf Coast tick (*Amblyomma maculatum*).

To sequence its mitochondrial genome, a female *A. maculatum* was procured from the Oklahoma State University Tick Rearing Facility. DNA was extracted from it using a DNeasy blood and tissue kit (Qiagen) and submitted to the Oregon Health & Science University’s Massively Parallel Sequencing Shared Resource. Sequencing libraries were prepared using the TruSeq DNA library kit (Illumina) and sequenced using a paired-end protocol on a HiSeq 2500 instrument (Illumina). FASTQ files were assembled from the base-called files using bcl2fastq v2.20 software (Illumina). This process yielded ∼180 million 100-bp read pairs. The reads were trimmed using Trimmomatic v0.39 (leading and trailing q-scores, ≥20; 5-bp sliding window q-scores, ≥25; length, ≥50 bp) and assembled into several thousand contigs using IDBA-UD v1.1.3 (6, 7). A single tick mitochondrial contig was identified among them using BLASTn v.2.6.0 (E value, <10^−15^) (8) and a database of all complete tick mitochondrial genome sequences publicly available in NCBI as of May 2017 (*n* = 47). All trimmed reads were mapped back to the mitochondrial contig as well as directly to the library of mitochondrial genome sequences, and the mapped reads were pooled and deduplicated, resulting in approximately 64,000 read pairs. These reads were assembled using IDBA-UD v1.1.3, yielding a linear 14,803-bp mitochondrial genome sequence (Table 1). PCR was used to close the genome and to validate the control region sequences; the PCR primer sequences are available on NCBI along with the genome sequence (GenBank accession number MW719251). The final assembly had an average sequencing coverage of 43×.

**TABLE 1:** *Amblyomma maculatum* mitochondrial genome characteristics

Characteristic	Value
GenBank accession no.	MW719251
AT content (%)	78.75
No. of protein coding genes	13
(+) strand	9
(−) strand	4
Start codon usage	
ATT	6
ATG	6
ATA	1
Stop codon usage	
TAA	10
T--	3
ENc^*a*^	35.19
No. of tRNAs	22
(+) strand	13
(−) strand	9
Position of putative regulatory elements	
Control region (ancestral)	8796–9100
Control region (derived)	14411–14745
Tick boxes	5827–5844
	6828–6845

a ENc, effective number of codons for all protein coding genes.

The *A. maculatum* mitochondrial genome sequence was annotated using MITOS rev.6b33f95 (9), and in order to perform a manual comparison of the mitochondrial gene arrangement, we reannotated the other 47 mitochondrial genome sequences using the same program. Similar to other Metastriata and Australasian Prostriata hard ticks, *A. maculatum*’s mitochondrial genome contains two copies of the control region, the putative regulatory region for mitochondrial replication and transcription (10) (Table 1). Previous reports indicate that the two regions evolved in synchrony, although the derived sequence is more variable in length (11, 12). In line with these findings, in *A. maculatum* the two control regions share an identical 275-bp segment, which accounts for most of the ancestral control region’s length, while the derived control region spans an additional 30 bp (Table 1). A putative posttranscriptional regulatory element known as the “tick box” is thought to be responsible for the extant Metastriata gene arrangement (10, 13). In *A. maculatum*, two tick box motifs occur as direct tandem repeats flanking a past relocation event (Table 1), whereas some other Metastriata species contain a third inverted tick box sequence (10, 13). It is unclear how prevalent this inverted repeat is among *Amblyomma* species; for instance, Amblyomma hebraeum contains the inverted repeat, but Amblyomma americanum does not (13).

**FIG 1:**
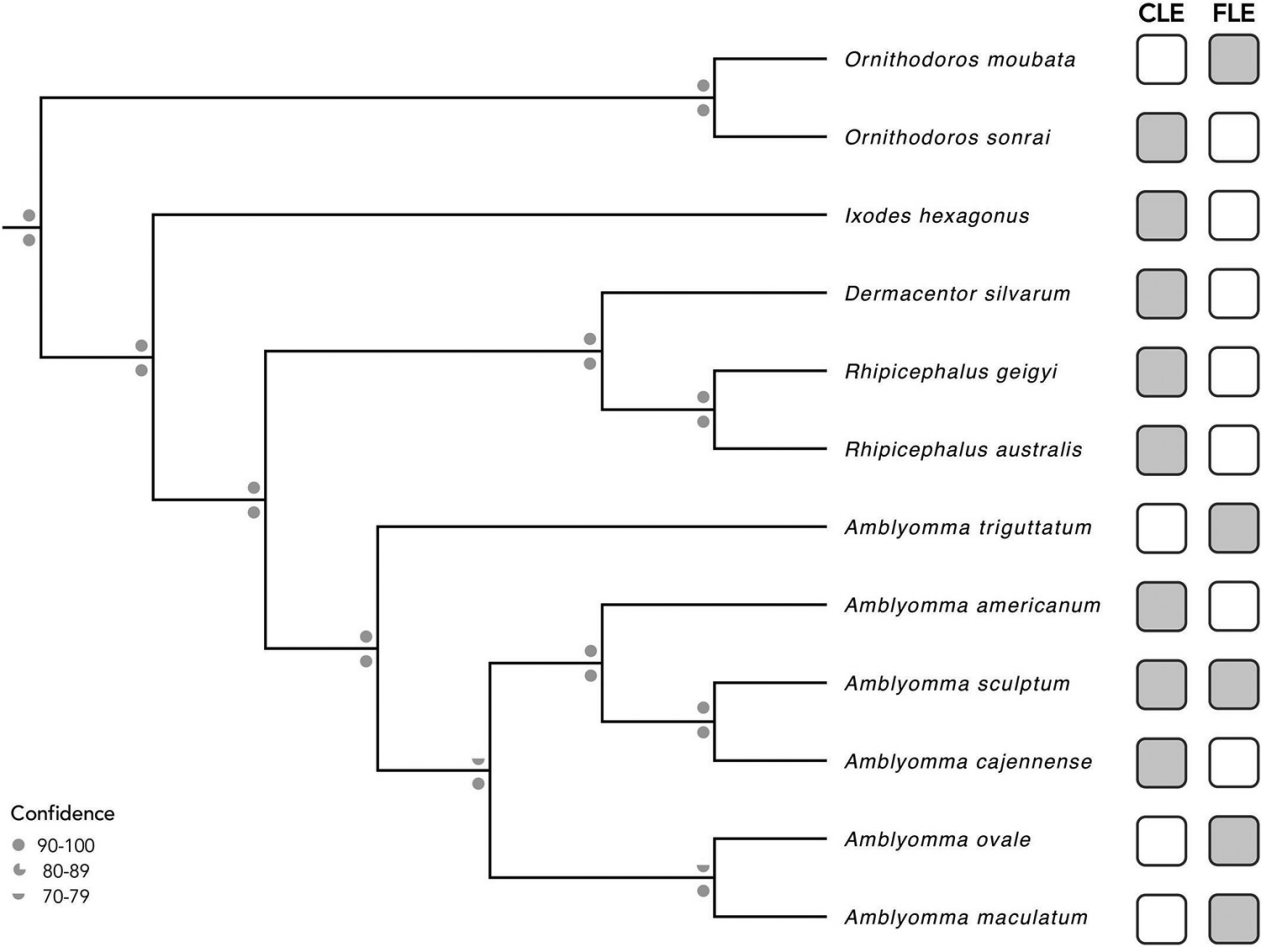
Distribution of primary endosymbionts in ticks. Maximum likelihood and Bayesian trees were generated using 13 mitochondrial protein coding gene sequences from 12 tick species with fully sequenced mitogenomes. The nucleotide sequences of the 13 protein coding genes were aligned individually using global MAFFT v7.475 (15) and then concatenated. GBlocks v0.91b (16) was used to cull ambiguously aligned regions, and jModelTest2 v2.1.10 (17) was used to select the appropriate model (GTR+I+G). The final tree is based on 10,488 nucleotide positions. The maximum likelihood tree was generated using RAxML v8.2.12, and the Bayesian tree was produced using MrBayes v3.2.7 (18, 19). Bootstrap support and posterior probabilities are depicted above and below the branch points, respectively. The gray and white boxes indicate the presence and absence, respectively, of endosymbionts in each tick (4, 5, 20).

To elucidate the evolutionary relationship of *A. maculatum* to other ticks, phylogenetic trees were generated using 10,488 bp of unambiguously aligned nucleotides. As illustrated in Fig. 1, the primary endosymbiont present in a tick is not determined solely by the tick phylogeny. CLEs are likely the ancestral tick endosymbiont, but newer CLEs and FLEs with better metabolic capabilities for supplying their hosts with nutrients have likely replaced the original CLEs (2, 14). In sum, the *A. maculatum* mitochondrial genome sequence presented here is expected to improve our understanding of mitochondrial noncoding regions and the evolutionary relationships between ticks and the endosymbionts they carry.

### Data availability.

The mitochondrial genome sequence for *Amblyomma maculatum* has been deposited in DDBJ/ENA/GenBank under the accession number MW719251. The genome assembly described in this paper is the first version, MW719251.1. The SRA records are available under accession number SRS8901492. The BioSample and BioProject accession numbers are SAMN19069232 and PRJNA728115, respectively.
